# Chromosome 16q22 variants in a region associated with cardiovascular phenotypes correlate with *ZFHX3* expression in a transcript-specific manner

**DOI:** 10.1186/s12863-014-0136-1

**Published:** 2014-12-24

**Authors:** Ruairidh I R Martin, W Andrew Owens, Michael S Cunnington, Bongani M Mayosi, Mauro Santibáñez Koref, Bernard D Keavney

**Affiliations:** Institute of Genetic Medicine, Newcastle University, Newcastle upon Tyne, UK; Division of Cardiothoracic Services, The James Cook University Hospital, South Tees Hospitals NHS Foundation Trust, Middlesbrough, UK; Hull and East Yorkshire NHS Trust, Hull, UK; Department of Medicine, University of Cape Town, Cape Town, South Africa; Institute of Cardiovascular Sciences, University of Manchester, Manchester, UK

**Keywords:** Expression QTL mapping, Trans-ethnic mapping, Atrial fibrillation, Genome-wide association study

## Abstract

**Background:**

The *ZFHX3* gene, located in Chromosome 16q22.3, codes for a transcription factor which is widely expressed in human tissues. Genome-wide studies have identified associations between variants within the gene and Kawasaki disease and atrial fibrillation. *ZFHX3* has two main transcripts that utilise different transcription start sites. We examined the association between genetic variants in the 16q22.3 region and expression of *ZFHX3* to identify variants that regulate gene expression.

**Results:**

We genotyped 65 single-nucleotide polymorphisms to tag genetic variation at the *ZFHX3* locus in two cohorts, 451 British individuals recruited in the North East of England and 310 mixed-ancestry individuals recruited in South Africa. Allelic expression analysis revealed that the minor (*A*) allele of rs8060701, a variant in the first intron of *ZFHX3*, was associated with a 1.16-fold decrease in allelic expression of both transcripts together, (p = 4.87e-06). The minor (*C*) allele of a transcribed variant, rs10852515, in the second exon of *ZFHX3* isoform *A* was independently associated with a 1.36-fold decrease in allelic expression of *ZFHX3 A* (p = 7.06e-31), but not overall *ZFHX3* expression. However, analysis of total gene expression of *ZFHX3* failed to detect an association with genotype at any variant. Differences in linkage disequilibrium between the two populations allowed fine-mapping of the locus to a 7 kb region overlapping exon 2 of *ZFHX3 A*. We did not find any association between *ZFHX3* expression and any of the variants identified by genome wide association studies.

**Conclusions:**

*ZFHX3* transcription is regulated in a transcript-specific fashion by independent *cis*-acting transcribed polymorphisms. Our results demonstrate the power of allelic expression analysis and trans-ethnic fine mapping to identify transcript-specific *cis*-acting regulatory elements.

**Electronic supplementary material:**

The online version of this article (doi:10.1186/s12863-014-0136-1) contains supplementary material, which is available to authorized users.

## Background

The chromosome 16q22.3 region contains the locus encoding the zinc-finger homeobox protein *ZFHX3* and has been shown to be associated with susceptibility to several cardiovascular disease phenotypes. Genome-wide association studies (GWAS) have identified associations between single-nucleotide polymorphisms (SNPs) in this region and atrial fibrillation (AF) [1-3], Kawasaki disease [4] and cardioembolic stroke [2,5]. The variants identified by GWAS are outside coding regions and so the associations are almost certainly mediated by influences on gene expression. Variants associated with cardiovascular disease span a region of 43 kb within the first intron of *ZFHX3*.

**Figure 1:**
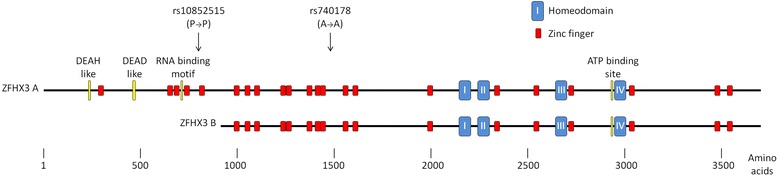
**ZFHX3 protein domains.** There are a large number of zinc finger and four homeobox nucleic acid binding domains. In the 915 amino acids unique to ZFHX3 A, there is a DEAD-like and a DEAH-like domain and an RNA binding motif. The positions of the transcribed synonymous SNPs rs10852515 and rs740178 are indicated by arrows.

*ZFHX3*, formerly known as *ATFB1*, is the only protein-coding gene within 500 kb of the GWAS hit SNPs. *ZFHX3* codes for a transcription factor (TF) which is widely expressed and is reported in all 16 tissues covered by the Body Map 2.0 project [6]. There are two known splice variants, *ZFHX3 A* and *ZFHX3 B*. The two transcripts have different promoter regions [7]. The *ZFHX3 A* transcript differs from the *B* isoform by the addition of 420 amino acids at the N-terminus (see Figure 1). Absence of *ZFHX3* expression and mutations near the ATP binding domain are associated with an increase in malignant activity in hepatocellular, gastric, breast and prostate carcinoma [8-11].

We have previously shown that the power of expression quantitative trait locus (eQTL) mapping using total levels of gene expression may be significantly limited by variation in *trans* acting factors such as age, gender or drugs. An alternative approach, which is specific for *cis* acting eQTLs, is to analyse relative expression of the two alleles of a transcript (allelic expression [aeQTL] mapping). Because both alleles are affected by *trans* acting influences such as age, disease status, medication etc., the allelic expression ratio (AER) is largely independent of them. We and others have shown that aeQTL mapping has a greater power to detect *cis* acting elements than eQTL mapping [12-14]. However, aeQTL mapping relies on there being one or more transcribed polymorphisms in a heterozygous state to determine the allelic origin of the transcripts.

The evidence for *cis*-acting eQTLs elements in *ZFHX3* thus far is conflicting. Associations between variants in the region, including GWAS hit SNPs, and gene expression have not been consistently identified in lymphoblastoid cell lines (LCLs) and no associations have been reported in primary tissues [15-17].

We used eQTL and aeQTL mapping to perform detailed fine-mapping of the association of SNPs at the 16q22.3 locus with expression of *ZFHX3*. We used DNA and RNA collected from peripheral blood cells in two cohorts of individuals recruited in the north-east of England (NE cohort) and South Africa (SA cohort). We identified multiple SNPs associated with allelic expression of *ZFHX3* whose effects were specific for each transcript.

## Methods

### Participants

Peripheral blood for DNA and RNA analysis was collected from anonymous adult volunteers in two cohorts: 310 South African mixed-ancestry blood donors and 451 hospital patients from north-east England. Of the NE cohort, 401 individuals were recruited at the time of cardiac catheterisation and 50 at the time of cardiac surgery. 66% were male and the median age was 68 years (range 22-88, lower quartile 59, upper quartile 74). Of the SA cohort 42% were male, with median age 20 years (range 17–60, lower quartile 19, upper quartile 23) [12].

### Ethics statement

The study complies with the Declaration of Helsinki. Informed consent was obtained from all participants and the study was approved by the Sunderland Local Research Ethics Committee and the University of Cape Town Faculty of Health Sciences Research Ethics Committee.

### Selection of transcribed SNPs for allelic expression analysis

Using the UCSC genome browser [18,19] transcribed SNPs with minor allele frequency (MAF) >0.05 were identified as suitable candidates for use in the allelic expression assays. Transcribed SNPs selected using these criteria were rs740178, in exon 8 of *ZFHX3 A* and exon 7 of *ZFHX3 B*, and rs10852515 in exon 2 of *ZFHX3 A*. There was no reported transcribed SNP which was specific for *ZFHX3 B*. Measurement of the allelic expression ratio (AER) using rs740178 allows assessment of both transcripts taken together. rs10852515 is not present in the spliced *ZFHX3 B* transcript and so measurement using rs10852515 allows assessment of the AER in *ZFHX3 A* alone (Figure 1).

### Selection of mapping SNPs

The lead GWAS hit SNP for Kawasaki disease is not in strong linkage disequilibrium (LD) with the AF hit SNPs, which are in strong LD with each other (Additional file 1: Table S1). Tag SNPs required to capture common variation in a core region of interest (Chr16: 72,801,786-73,107,534) based on HapMap CEU data were selected using HaploView 4.0 tagger software. We used the following parameters: minimum minor allele frequency 0.05, pairwise tagging, r^2^ threshold >0.8. SNPs previously reported to be associated with disease phenotypes were force-included in the SNP selection process [1-4,20]. Details of included SNPs are shown in Additional file 1: Tables S2-S4.

### Genotyping and measurement of expression

Genotyping and measurement of allelic expression ratios was performed by MALDI-TOF mass spectrometry (Sequenom). Total gene expression was measured using real-time PCR, according to MIQE guidelines [21]. Full details are included in the supplementary methods.

### Statistical analyses

The association between total expression, as measured by real time PCR, and each of the SNPs was assessed using linear regression of the log transformed normalised expression values on the genotype, assuming no dominance or interactions between the effects of different SNPs. PCR plate was included as a categorical variable.

Genotype phase was estimated for each cohort separately using the BEAGLE v3.3.2 Genetic Analysis Software Package [22]. LD was calculated using phased genotypes in HaploView v4.2 and haplotype blocks were identified using the confidence interval algorithm [23,24]. We analysed allelic expression ratios and estimated the proportion of variance that was due to *cis-*acting effects using the approaches that we published previously [12]. The analyses in the NE cohort were replicated in the SA cohort and the results compared to allow trans-ethnic fine mapping of associations. In order to provide increased power, the results from each cohort were meta-analysed using Fisher’s method.

For both total and allelic expression multiple testing, corrected p-values were calculated as the family-wise error rate (FWER) using Holm’s correction for the 65 SNPs tested. Holm’s procedure is more efficient than the Bonferroni method of correction for multiple association testing and so is less conservative, without an increase in type I error [25]. Associations with a corrected p-value below a threshold of 0.05 were considered significant. From our allelic and total expression data we also estimated the proportion of the total expression variance that is due to *cis*-acting effects using previously published methods [12].

## Results

After quality control, we measured total expression of *ZFHX3* in peripheral blood from 366 individuals in the NE cohort. Allelic expression was assessed for both transcripts taken together using the transcribed SNP rs740178 in 132 individuals in both cohorts and for the A transcript alone using the transcribed SNP rs10852515 in 152 individuals in both cohorts. We selected 65 SNPs that tag the common variation in the region, specifically including SNPs with previously reported phenotypic associations. The results of allelic expression mapping were compared with conventional mapping using total expression in the samples. The results are summarised in Additional file 1: Tables S2-S4.

### Inter-individual variation in expression

Total *ZFHX3* expression levels between individuals varied up to 9.64-fold. Expression ratios at individual transcribed markers also showed inter-individual variation, the range of the ratio of the minor:major alleles was 0.71:1– 1.65:1 for both transcripts taken together and 0.39:1– 1.48:1 for transcript A alone, indicating that *ZFHX3* expression is regulated by factors which differ between individuals and that there was greater inter-individual variability in the AER of transcript A. Plots of the log allelic expression ratios and log_2_ total expression values for each individual are shown in Additional file 1: Figure S1. We estimated 3.7% of the variance to be due to *cis*-acting effects.

### Analysis of total expression

**Figure 2:**
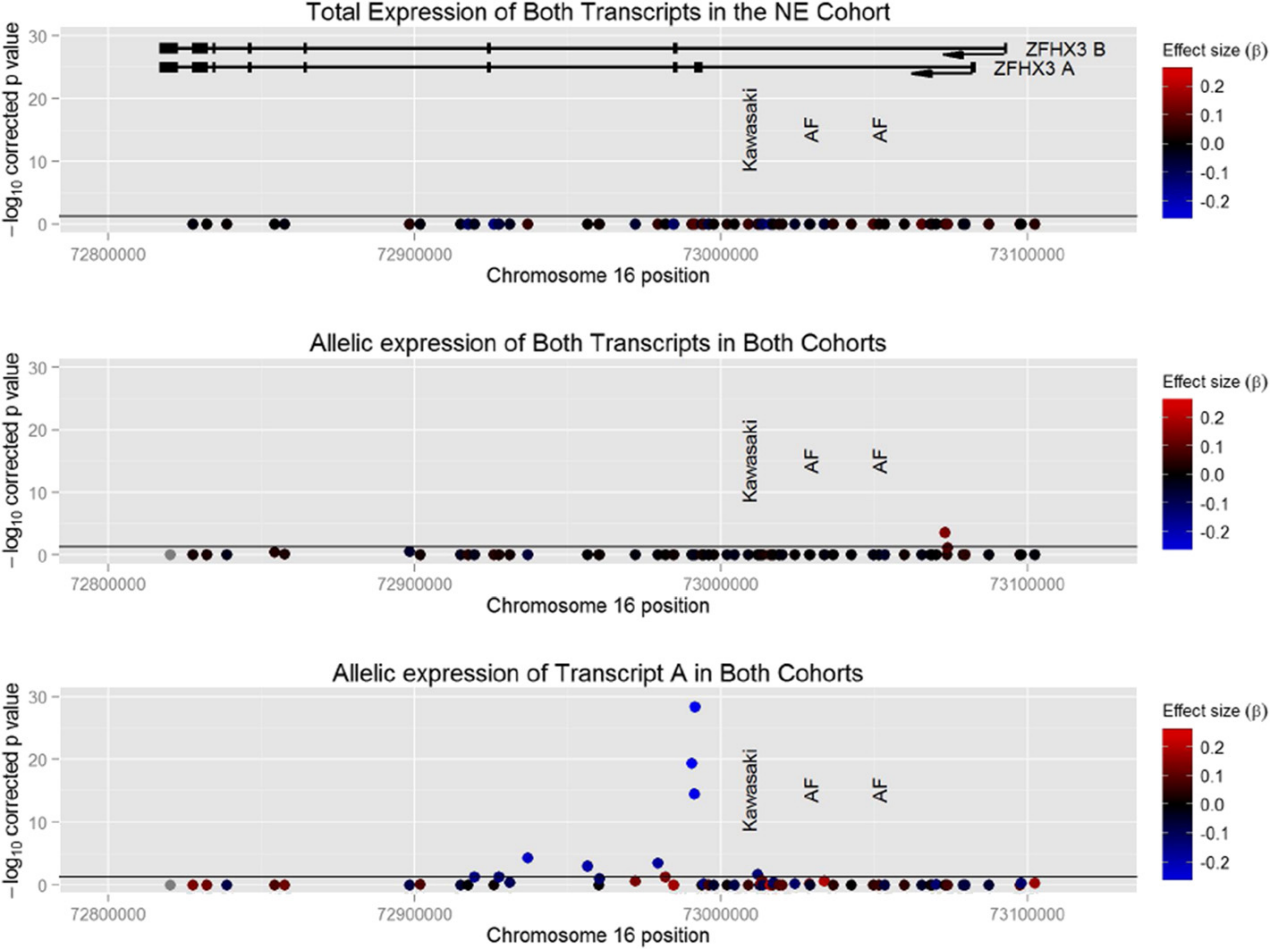
**Associations between SNP genotype and gene expression.** SNPs are plotted with chromosomal position on the x axis and –log p value on the y axis. The horizontal line represents the significance threshold (p = 0.05). Individual points are coloured to indicate effect size (β). No SNP is significantly associated with whole gene expression. A single SNP, rs8060701, is significantly associated with allelic expression of both transcripts together. 11 SNPs are associated with allelic expression of the A transcript. The cartoon at top shows the position of transcripts A and B of ZFHX3. Both isoforms are transcribed in the reverse direction, arrow.

No significant associations were detected between SNP genotype and total expression of *ZFHX3* in the NE cohort, as assessed by qPCR (Figure 2 and Additional file 1: Table S2). As no associations were seen replication was not performed in the SA cohort.

### Comparison of patterns of linkage disequilibrium between populations

Patterns of LD in the two populations are shown in Additional file 1: Figure S2. The pattern of LD and the haplotype blocks differed significantly between the two populations. We analysed aeQTLs in both populations separately to determine the effects of different population haplotype structures on aeQTLs. We subsequently performed a meta analysis of the results in both cohorts, increasing the power to detect smaller cis-acting effects. Raw and corrected p values are shown in Additional file 1: Tables S3 and S4.

### Analysis of allelic expression of both *A* and *B* transcripts

aeQTL mapping of both transcripts together identified a single significantly associated SNP, rs8060701 in the NE cohort (p = 1.29e-04) (Additional file 1: Table S3). This was replicated in the SA cohort (p = 2.82e-03). When both data sets were combined, the minor (*A*) allele was associated with a 1.17-fold decrease in expression of both *ZFHX3* transcripts combined (p = 4.87e-06, corrected p = 3.17e-04). After correcting for the effect of rs8060701, no further associations were seen in any cohort.

### Analysis of allelic expression of *ZFHX3 A*

aeQTL mapping of transcript A in the NE cohort revealed several strongly associated SNPs (Figure 2 and Additional file 1: Table S4). The most strongly associated SNP was the transcribed SNP, rs10852515 (p = 7.47e-15). This association was strongly replicated in the SA cohort (p = 1.12e-16). In the combined data set, the minor (*C*) allele was associated with a 1.35-fold decrease in *ZFHX3 A* expression (p = 9.61e-31, corrected p = 6.25e-29). After correction for the effects of rs10852515, no other variants were independently associated with expression. The aeQTL SNP associated with expression of both transcripts, rs8060701, was not independently associated with *ZFHX3 A* expression, rs10852515 and rs8060701 were not in significant LD in the SA cohort (r^2^ = 0) or the NE cohort (r^2^ = 0.005).

**Figure 3:**
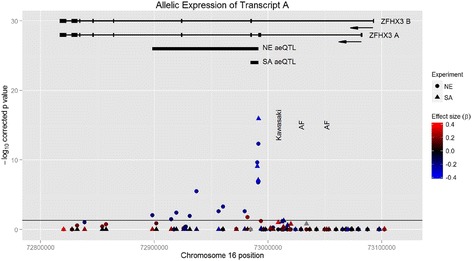
**Comparison of results for ZFHX3 A expression in the SA and NE cohorts.** SNPs are plotted with chromosomal position on the x axis and strength of association n the y axis. Colour indicates effect size. Results from the NE and SA cohorts are shown as squares and triangles respectively. The regions of significant association with ZFHX3 A expression in each cohort are shown as black bars.

The pattern of association between *ZFHX3 A* expression and SNP genotype differed between the two populations. After correction for multiple testing, the aeQTL in the NE cohort included 12 significantly associated SNPs in a 93 kb region. By contrast, in the SA cohort the aeQTL was confined to three SNPs in a 7 kb region (Figure 3). In both cohorts, the significantly associated variants were in LD with the SNP with the most significant p-value, rs10852515. The log p value for the association was significantly associated with the degree of LD (r^2^) with rs10852515 in both cohorts, confirming that the differences between associations in the two cohorts were a result of differing patterns of LD (Additional file 1: Figure S3).

### Associations between GWAS significant variants and *ZFHX3* expression

The Kawasaki disease-associated SNP, rs7199343 and the AF-associated SNPs rs7193373 and rs2106261 were not significantly associated with *ZFHX3 A* expression (corrected p > 0.05 in all cases).

## Discussion

We have shown that multiple SNPs in the *ZFHX3* region are significantly associated with *ZFHX3* expression in whole blood in a transcript specific manner. We have identified an aeQTL regulating *ZFHX3* isoform A expression, demonstrating that allelic expression analysis is a powerful approach to investigation of transcript-specific gene expression. Additionally we have confirmed in peripheral blood the association between rs8060701 and expression of both transcripts in LCLs; this is the first demonstration of *cis-*acting influences on *ZFHX3* expression in primary human tissue.

Expression levels of *ZFHX3* vary significantly between individuals and *cis-*acting factors account for only 3.7% of that variance. *ZFHX3* is involved in the cellular response to genotoxic and oxidative stress [26] and its expression is known to be influenced by a large number of *trans* acting factors, including cytokine levels, viral infection, tissue injury and drug treatment [27].

A number of SNPs were associated with ZFHX3 expression. However after correcting for the effect of rs10852515, no other significant associations remained, which is consistent with the transcribed SNP itself being either the functional SNP or in strong LD with an untyped functional SNP. The use of the Cape mixed-ancestry cohort allowed much finer localisation of the aeQTL from a 93 kb to a 7 kb region. This study therefore demonstrates the power of trans-ethnic fine mapping combined with allelic expression analysis to fine-map aeQTLs within regions associated with gene expression.

In the lymphoblastoid cell line (LCL) arm of the MuTHER study, a microarray-based genome-wide analysis of gene expression in skin, adipose tissue and LCLs in a cohort of UK twins, an association was found between rs8060701 and ZFHX3 expression (n = 856, p = 8.27e-05) [16,28]. This association was not found in the tissue arms of the MuTHER study or in the GenCord study but has been demonstrated here. Our findings, therefore, confirm that this association is present in primary human tissue. The Illumina probe used in the MuTHER and GenCord studies is not transcript-specific and so would not have allowed detection of transcript-specific eQTLs.

Changes in the relative expression of the two isoforms of *ZFHX3* has been shown to determine the rate of differentiation of C2C12 myoblasts, the *B* transcript accelerating myogenic differentiation and the *A* isoform resulting in persistence of an undifferentiated phenotype [29]. This suggests that transcript-specific regulation of *ZFHX3* expression may have an important role in control of differentiation and cellular proliferation. Transcript-specific regulation of gene expression has been associated with other diseases including schizophrenia, affective disorders and hepatocellular carcinoma [30,31].

We found no association between *ZFHX3* expression and genotype at those SNPs associated with Kawasaki disease or AF. *ZFHX3* was selected as a candidate gene for the mechanism of action of the GWAS hit SNPs as the lead SNPs lie in the first intron of the gene and no other genes lie in the region of association. It is possible, however, that the mechanism of action of these SNPs is mediated through a distant transcript. An alternative explanation for the lack of association is that the effects of the GWAS lead SNPs on gene expression are tissue specific and that there are *cis*-acting eQTLs in atrial or other disease-relevant tissues that are not active in peripheral blood.

### Limitations

**Figure 4:**
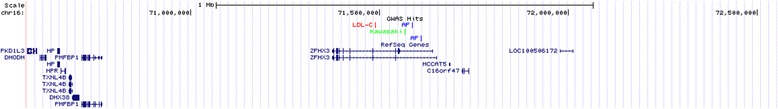
**The ZFHX3 locus.** Refseq genes within 1 MB of the identified GWAS SNPs are shown. There is a large region with relatively few protein-coding genes which extends 800 Mb from the risk polymorphisms, highlighting the relevance of ZFHX3 as a candidate gene.

Our results demonstrate that the SNPs rs8060701 and rs10852515 are associated with ZFHX3 expression. From our data, however, it is not possible to determine whether the lead SNPs are the functional variants or are simply in strong LD with the true functional variants. Further studies are required to identify the exact mechanisms by which the variants regulate expression. We found no association between the GWAS hit SNPs at this locus and ZFHX3 expression. As there are very few other genes in this region that might be responsible for the GWAS associations (Figure 4), it is possible that there are eQTLs for ZFHX3 in other tissues that we have been unable to detect in peripheral blood.

## Conclusions

We have identified two independent *cis*-acting regulatory elements in *ZFHX3* which act in a transcript-specific fashion. We have discovered a transcribed polymorphism which regulates *ZFHX3* isoform *A* expression, using trans-ethnic fine mapping to localise the functional SNP. We have also confirmed that variants in the first intron of *ZFHX3* that are associated with overall *ZFHX3* expression in LCLs are also associated with expression in peripheral blood. These findings confirm the increased power of the aeQTL approach over eQTL mapping to identify *cis*-acting variants and additionally demonstrate the usefulness of this technique in identifying transcript-specific regulatory elements.

## Additional file

Additional file 1:
**Supplementary Methods and Results.**

## Abbreviations

aeQTL: Allelic expression quantitative trait locus
AER: Allelic expression ratio
AF: Atrial fibrillation
eQTL: Expression quantitative expression locus
GWAS: Genome-wide association study
LCL: Lymphoblastoid cell line
LD: Linkage disequilibrium
LDL-C: Low density lipoprotein cholesterol
MAF: Minor allele frequency
NE: North-east England
SA: South African
SNP: Single nucleotide polymorphism
