# Supplementary material for: Chromosome 16q22 variants in a region associated with cardiovascular phenotypes correlate with ZFHX3 expression in a transcript-specific manner
Source: BMC Genet. 2014 Dec 24;15:136. doi: 10.1186/s12863-014-0136-1 (PMC4301889; doi:10.1186/s12863-014-0136-1)
Supplement: Additional file 1: — Supplementary Methods and Results. [file 12863_2014_136_MOESM1_ESM.pdf]

Supplementary Data

Chromosome 16q22 variants associated with low-density lipoprotein cholesterol levels correlate with ZFHX3 expression in a transcript-specific manner

Supplementary Methods

DNA and RNA extraction and cDNA analysis

8ml of blood was collected in EDTA at the time of recruitment. DNA was extracted using standard methods.

RNA was extracted from 2.5ml of blood collected using the PAXgene system (Qiagen) following the manufacturer's protocol and contaminating DNA was removed using the DNAfree DNase kit (Ambion). For gene expression assays approximately 2μg of RNA was reverse transcribed in a 20μl reaction using the SuperScript VILO cDNA synthesis kit (Invitrogen) and diluted to a final concentration of 25μg RNA template/μl with water.

RNA integrity was determined on a random subset of samples using the RNA 6000 Pico LabChip kit (Agilent, USA) on the Agilent 2100 bioanalyser, following the manufacturer's standard protocol. The median RNA integrity number was 7.85, indicating sufficient quality for PCR-based assay methods[1].

Genotyping

Multiplex SNP genotyping was performed by primer extension and MALDI-TOF mass spectrometry using iPLEX Gold SNP technology (Sequenom). SNP assays were designed using Sequenom's MassARRAY Assay Design v3.0 Software (multiplex details and primer sequences are available in Table S5). PCR was performed using 25ng in a 7μl reaction volume for 35 cycles using standard iPLEX methodology. Spectra were analysed using MassARRAY Typer v4.0 Software (Sequenom). Spectra and plots were manually reviewed and auto-calls were manually adjusted if required. Assay quality control was performed using 92 CEPH samples to assess genotype concordance with HapMap genotypes. Individual samples with low genotype call rates (<95%) and SNP assays with poor quality spectra/cluster plots were excluded. Correspondence to Hardy-Weinberg proportions was checked for each SNP.

Measurement of allelic expression ratios

Allelic expression ratios were determined in the NE and SA cohorts. Assays for the measurement of allelic expression were identical to those used for genotyping rs740178 and rs10852515 as the amplicons for each are wholly contained within a single exon. Primer sequences are shown in Table S6. Contamination with genomic DNA was assessed in a subset of 12 samples by performing PCR without the reverse transcription step and running the products on an agarose gel. No genomic DNA contamination was detected.

Quantification of the allelic expression ratio was performed by primer extension and MALDI-TOF spectrometry using iPLEX Gold with identical parameters to the genotyping assay. Spectra were analysed using MassARRAY Typer v4.0 Software (Sequenom) and allelic expression ratios were estimated as the ratios of the area under the peak representing allele 1 to that representing allele 2. Measurements were performed in four replicates using 25ng cDNA template. Results from the amplification of genomic DNA were used as an equimolar reference to normalise the cDNA values. The gDNA ratios were relatively homogenous with little inter-individual variability compared to cDNA ratios (Figure S1). AER values were excluded where the standard error of the technical replicates was high (>0.5). Outliers were excluded using Grubb's test.

Relative quantification of total gene expression using real-time PCR

Quantification of total gene expression was performed in the NE cohort only. Real time PCR reactions were performed according to the MIQE guidelines[2]. Assays were performed using TaqMan gene

expression probes and reagents (Life Technologies) and run on a 7900HT Real Time PCR System (Applied Biosystems). A commercially available validated VIC-labelled assay (Hs00199344_m1) for ZFHX3, which overlies the exon 5-6 boundary for transcript A and the exon 4-5 boundary for transcript B, was used. Commercially available primer-limited VIC-labelled assays were used for three reference genes, RN18S1 (Hs03928990_g1), ACTB (Hs99999903_m1) and GAPDH (Hs03929097_g1) (Life Technologies, USA). PCR was performed in four replicates according to the manufacturer's protocol using 25ng of cDNA template in a 15µl reaction volume.

Relative total expression was analysed using the comparative cycle threshold (Ct) method. Ct values for the target gene were normalised to the mean Ct value of the three reference genes[3]. Standard errors of measurements for allelic and total expression analyses are shown in Table S7. Delta Ct values were excluded where the standard error of the technical replicates was high (>0.5). Outliers were excluded using Grubb's test.

Supplementary References

1. Fleige S, Pfaffl MW: **RNA integrity and the effect on the real-time qRT-PCR performance**. *Mol Aspects Med* 2006, **27**(2-3):126-139.
2. Bustin SA, Benes V, Garson JA, Hellemans J, Huggett J, Kubista M, Mueller R, Nolan T, Pfaffl MW, Shipley GL *et al*: **The MIQE guidelines: minimum information for publication of quantitative real-time PCR experiments**. *Clin Chem* 2009, **55**(4):611-622.
3. Vandesompele J, De Preter K, Pattyn F, Poppe B, Van Roy N, De Paepe A, Speleman F: **Accurate normalization of real-time quantitative RT-PCR data by geometric averaging of multiple internal control genes**. *Genome biology* 2002, **3**(7):RESEARCH0034.

Table S1. LD between GWAS hit SNPs in the NE and SA cohorts.

NE Cohort

SNP 1	GWAS hit	SNP 2	GWAS hit	D'	r^2	Distance
rs16971384	LDL-C	rs7199343	Kawasaki	0.069	0.001	77939
rs16971384	LDL-C	rs7193343	AF	0.151	0.012	98075
rs16971384	LDL-C	rs2106261	AF	0.091	0.005	120535
rs7199343	Kawasaki	rs7193343	AF	0.529	0.033	20136
rs7199343	Kawasaki	rs2106261	AF	0.228	0.006	42596
rs7193343	AF	rs2106261	AF	0.854	0.714	22460

SA Cohort

SNP 1	GWAS hit	SNP 2	GWAS hit	D'	r^2	Distance
rs16971384	LDL-C	rs7199343	Kawasaki	0.554	0.036	77939
rs16971384	LDL-C	rs7193343	AF	0.016	0	98075
rs16971384	LDL-C	rs2106261	AF	0.028	0	120535
rs7199343	Kawasaki	rs7193343	AF	0.556	0.015	20136
rs7199343	Kawasaki	rs2106261	AF	0.273	0.003	42596
rs7193343	AF	rs2106261	AF	0.69	0.371	22460

Table S2. Effect size and association strength for whole gene expression. The uncorrected and corrected p values and effect sizes are given for each typed SNP.

SNP	Position	Importance	NE Cohort (n=366)		
			Effect size (β)	p	corrected p
rs117951282	72820328		-	-	-
rs699444	72827758		-0.02	7.79E-01	NS
rs740178	72832135	Transcribed	0.01	9.18E-01	NS
rs12929452	72838680		0.01	8.26E-01	NS
rs2266943	72854307		0.00	9.72E-01	NS
rs8058014	72857544		-0.02	7.51E-01	NS
rs1476646	72898348		0.05	2.83E-01	NS
rs4788668	72901874		-0.02	6.98E-01	NS
rs4788671	72915085		-0.01	7.22E-01	NS
rs6499594	72917601		-0.11	7.31E-02	NS
rs16971366	72919582		-0.03	4.84E-01	NS
rs9940310	72925982		-0.14	5.58E-02	NS
rs9925261	72927533		-0.07	9.76E-02	NS
rs16971384	72931085		-0.04	4.37E-01	NS
rs4788482	72937079		0.06	3.40E-01	NS
rs4788679	72956544		0.01	9.07E-01	NS
rs9936884	72960230		0.04	5.67E-01	NS
rs4788488	72960283		0.02	6.97E-01	NS
rs8055870	72972090		-0.06	1.89E-01	NS
rs6499600	72979374		0.06	1.54E-01	NS
rs13336412	72981949		0.00	9.10E-01	NS
rs2228200	72984668		-0.11	1.96E-01	NS
rs2106258	72990553		0.06	2.46E-01	NS
rs2157786	72991286		0.06	1.94E-01	NS
rs10852515	72991660	Transcribed	0.10	1.23E-01	NS
rs7193297	72993831		-0.03	3.84E-01	NS
rs7404992	72994419		0.06	4.61E-01	NS
rs756717	72996162		-0.07	1.32E-01	NS
rs4788683	72997747		0.01	8.10E-01	NS
rs9921395	73001957		0.03	4.35E-01	NS
rs12445932	73004432		0.00	9.96E-01	NS
rs7199343	73009024	Kawasaki GWAS	0.07	1.31E-01	NS
rs11075954	73012164		-0.05	2.81E-01	NS
rs2040508	73012685		0.08	1.04E-01	NS
rs16971456	73013036		0.01	8.63E-01	NS
rs9930445	73013482		0.03	5.50E-01	NS
rs4788684	73013633		-0.09	3.63E-02	NS
rs16971464	73016143		-0.15	6.39E-02	NS
rs16971465	73017061		-0.06	3.83E-01	NS
rs4788489	73017118		0.01	8.97E-01	NS

rs16971474	73019004		0.02	6.50E-01	NS
rs11640106	73020116		0.02	5.76E-01	NS
rs1858800	73024276		-0.04	3.12E-01	NS
rs756720	73028921		-0.07	1.81E-01	NS
rs7193343	73029160	AF GWAS	-0.02	7.00E-01	NS
rs11075958	73033869		-0.06	2.49E-01	NS
rs8056528	73036633		0.04	3.84E-01	NS
rs719353	73042551		0.03	3.99E-01	NS
rs4788689	73049830		0.09	1.42E-01	NS
rs2106261	73051620	AF GWAS	0.02	7.14E-01	NS
rs11863932	73053579		0.01	9.26E-01	NS
rs1548373	73059861		0.01	8.81E-01	NS
rs4788692	73065656		0.10	1.71E-01	NS
rs12373097	73068515		-0.05	3.20E-01	NS
rs8057081	73068977		0.03	5.44E-01	NS
rs4788696	73070310		-0.01	9.01E-01	NS
rs8060701	73073289		0.03	6.78E-01	NS
rs9940321	73073808		0.03	5.21E-01	NS
rs9940520	73074012		0.08	1.90E-01	NS
rs11641701	73079212		0.00	9.36E-01	NS
rs7204751	73079683		-0.02	7.26E-01	NS
rs4788697	73087494		0.05	2.47E-01	NS
rs8052905	73097663		0.05	6.29E-01	NS
rs739414	73097956		0.00	9.92E-01	NS
rs8051826	73102456		0.05	4.89E-01	NS

Table S3. Effect size and association strength for allelic expression of both transcripts together. The uncorrected and corrected p values and effect sizes are given for each typed SNP.

SNP	Position	Importance	NE Cohort (n=106)			SA Cohort (n=26)			Both Cohorts (n=132)		
			Effect size (β)	p	corrected p	Effect size (β)	p	corrected p	Effect size (β)	p	corrected p
rs117951282	72820328		-	-	-	-	-	-	-	-	NS
rs699444	72827758		0.03	5.22E-02	NS	0.01	7.16E-01	NS	0.03	4.66E-02	NS
rs740178	72832135	Transcribed	0.03	2.86E-02	NS	0.03	2.58E-01	NS	0.03	1.44E-02	NS
rs12929452	72838680		-0.04	1.19E-01	NS	0.00	9.33E-01	NS	-0.03	1.32E-01	NS
rs2266943	72854307		0.03	2.96E-02	NS	0.05	4.30E-02	NS	0.04	6.57E-03	NS
rs8058014	72857544		0.03	4.67E-02	NS	0.04	7.81E-02	NS	0.03	1.33E-02	NS
rs1476646	72898348		-0.05	8.63E-03	5.43E-01	-0.04	3.80E-01	NS	-0.05	4.86E-03	NS
rs4788668	72901874		0.03	1.06E-01	NS	0.00	8.57E-01	NS	0.02	1.49E-01	NS
rs4788671	72915085		-0.04	6.22E-02	NS	0.00	9.91E-01	NS	-0.03	7.21E-02	NS
rs6499594	72917601		0.06	1.33E-02	8.27E-01	-0.03	3.80E-01	NS	0.04	5.79E-02	NS
rs16971366	72919582		-0.02	4.58E-01	NS	-0.06	8.82E-02	NS	-0.03	1.91E-01	NS
rs9940310	72925982		0.06	2.42E-02	NS	-0.04	3.43E-01	NS	0.04	7.05E-02	NS
rs9925261	72927533		0.03	1.23E-01	NS	0.00	9.52E-01	NS	0.03	1.44E-01	NS
rs16971384	72931085		0.02	2.58E-01	NS	0.00	9.98E-01	NS	0.02	2.80E-01	NS
rs4788482	72937079		-0.06	6.34E-02	NS	-0.14	3.02E-02	NS	-0.08	1.58E-02	NS
rs4788679	72956544		0.01	6.94E-01	NS	-0.07	5.10E-02	NS	-0.01	7.02E-01	NS
rs9936884	72960230		0.02	3.85E-01	NS	0.08	5.05E-02	NS	0.03	1.47E-01	NS
rs4788488	72960283		0.01	4.69E-01	NS	0.01	8.41E-01	NS	0.01	4.54E-01	NS
rs8055870	72972090		-0.02	3.81E-01	NS	0.01	8.68E-01	NS	-0.01	4.29E-01	NS
rs6499600	72979374		0.01	8.13E-01	NS	-0.07	2.06E-02	NS	-0.01	5.52E-01	NS
rs13336412	72981949		-0.01	6.99E-01	NS	0.06	7.64E-02	NS	0.01	8.42E-01	NS
rs2228200	72984668		0.03	4.42E-01	NS	-0.19	9.97E-02	NS	-0.01	6.95E-01	NS
rs2106258	72990553		-0.02	4.80E-01	NS	-0.01	7.78E-01	NS	-0.02	4.45E-01	NS
rs2157786	72991286		-0.01	6.94E-01	NS	0.03	2.67E-01	NS	0.00	9.17E-01	NS
rs10852515	72991660	Transcribed	-0.04	1.77E-01	NS	0.00	9.23E-01	NS	-0.03	2.09E-01	NS
rs7193297	72993831		0.04	1.27E-01	NS	0.02	5.38E-01	NS	0.03	1.01E-01	NS
rs7404992	72994419		-0.01	8.49E-01	NS	0.06	1.41E-01	NS	0.01	6.40E-01	NS
rs756717	72996162		-0.02	4.61E-01	NS	-0.02	5.90E-01	NS	-0.02	3.79E-01	NS
rs4788683	72997747		0.01	4.60E-01	NS	-0.01	8.36E-01	NS	0.01	5.46E-01	NS
rs9921395	73001957		-0.04	7.91E-02	NS	0.03	3.49E-01	NS	-0.02	1.82E-01	NS
rs12445932	73004432		-0.03	2.31E-01	NS	-0.03	6.04E-01	NS	-0.03	1.89E-01	NS
rs7199343	73009024	Kawasaki GWAS	-0.02	2.71E-01	NS	-0.01	8.78E-01	NS	-0.02	2.62E-01	NS
rs11075954	73012164		0.02	2.44E-01	NS	-0.03	3.57E-01	NS	0.01	4.93E-01	NS
rs2040508	73012685		-0.01	5.97E-01	NS	0.00	9.28E-01	NS	-0.01	6.58E-01	NS
rs16971456	73013036		-0.03	3.67E-01	NS	0.04	3.65E-01	NS	-0.01	6.27E-01	NS
rs9930445	73013482		-0.03	1.71E-01	NS	0.02	5.24E-01	NS	-0.02	3.48E-01	NS
rs4788684	73013633		0.03	8.34E-02	NS	-0.02	5.30E-01	NS	0.02	1.82E-01	NS
rs16971464	73016143		0.05	1.77E-01	NS	0.03	7.11E-01	NS	0.05	1.55E-01	NS
rs16971465	73017061		0.01	7.14E-01	NS	-0.03	4.93E-01	NS	0.00	9.45E-01	NS
rs4788489	73017118		0.01	8.13E-01	NS	0.00	8.77E-01	NS	0.00	7.83E-01	NS

rs16971474	73019004		-0.02	5.46E-01	NS	0.03	3.58E-01	NS	-0.01	8.81E-01	NS
rs11640106	73020116		-0.02	4.14E-01	NS	-0.01	7.04E-01	NS	-0.02	3.66E-01	NS
rs1858800	73024276		0.02	3.18E-01	NS	-0.09	1.99E-02	NS	0.00	8.26E-01	NS
rs756720	73028921		0.00	9.95E-01	NS	-0.01	7.82E-01	NS	0.00	9.23E-01	NS
rs7193343	73029160	AF GWAS	-0.02	4.84E-01	NS	0.07	8.61E-02	NS	0.00	9.39E-01	NS
rs11075958	73033869		-0.01	6.23E-01	NS	-0.04	5.13E-01	NS	-0.02	5.08E-01	NS
rs8056528	73036633		-0.01	7.83E-01	NS	0.02	5.47E-01	NS	0.00	9.98E-01	NS
rs719353	73042551		0.01	7.48E-01	NS	-0.05	8.62E-02	NS	-0.01	7.73E-01	NS
rs4788689	73049830		-0.03	2.62E-01	NS	0.03	5.19E-01	NS	-0.02	4.28E-01	NS
rs2106261	73051620	AF GWAS	-0.01	7.10E-01	NS	0.01	9.09E-01	NS	-0.01	7.40E-01	NS
rs11863932	73053579		-0.05	2.94E-01	NS	-0.03	6.95E-01	NS	-0.04	2.65E-01	NS
rs1548373	73059861		0.02	3.68E-01	NS	0.02	5.06E-01	NS	0.02	2.81E-01	NS
rs4788692	73065656		-0.06	7.31E-02	NS	0.05	1.44E-01	NS	-0.04	3.97E-01	NS
rs12373097	73068515		-0.02	5.79E-01	NS	-0.07	1.96E-02	NS	-0.03	1.47E-01	NS
rs8057081	73068977		-0.01	7.74E-01	NS	0.04	4.01E-01	NS	0.00	9.73E-01	NS
rs4788696	73070310		0.00	9.45E-01	NS	0.02	6.04E-01	NS	0.00	9.17E-01	NS
rs8060701	73073289		**0.17**	**1.29E-04**	**8.40E-03**	0.12	2.82E-03	1.83E-01	**0.16**	**4.87E-06**	**3.17E-04**
rs9940321	73073808		0.03	2.87E-01	NS	0.06	4.20E-02	NS	0.03	7.40E-02	NS
rs9940520	73074012		0.11	3.63E-03	2.33E-01	0.06	6.89E-02	NS	0.10	1.20E-03	NS
rs11641701	73079212		0.01	7.58E-01	NS	0.04	1.92E-01	NS	0.01	4.18E-01	NS
rs7204751	73079683		0.05	1.24E-01	NS	0.06	9.02E-02	NS	0.05	3.67E-02	NS
rs4788697	73087494		-0.02	2.94E-01	NS	0.02	4.77E-01	NS	-0.02	4.83E-01	NS
rs8052905	73097663		0.03	5.60E-01	NS	0.06	1.94E-01	NS	0.03	2.65E-01	NS
rs739414	73097956		0.00	9.88E-01	NS	0.02	6.47E-01	NS	0.00	8.73E-01	NS
rs8051826	73102456		-0.01	6.36E-01	NS	0.02	7.46E-01	NS	-0.01	7.03E-01	NS

Table S4. Effect size and association strength for allelic expression of transcript A. The uncorrected and corrected p values and effect sizes are given for each typed SNP.

SNP	Position	Importance	NE Cohort (n=66)			SA Cohort (n=86)			Both Cohorts (n=152)		
			Effect size (β)	p	corrected p	Effect size (β)	p	corrected p	Effect size (β)	p	corrected p
rs117951282	72820328		-	-	-	0.36	2.09E-01	NS	-	-	NS
rs699444	72827758		0.15	2.67E-02	NS	0.06	8.06E-01	NS	0.10	1.04E-01	NS
rs740178	72832135	Transcribed	0.18	5.49E-03	2.69E-01	0.00	1.00E+00	NS	0.08	3.41E-02	NS
rs12929452	72838680		-0.16	1.70E-03	8.83E-02	0.22	6.93E-02	NS	0.05	1.15E-01	NS
rs2266943	72854307		0.17	7.39E-03	3.55E-01	-0.02	8.28E-01	NS	0.06	5.10E-02	NS
rs8058014	72857544		0.19	3.53E-03	1.76E-01	-0.02	8.73E-01	NS	0.07	2.63E-02	NS
rs1476646	72898348		**-0.19**	**1.55E-04**	**8.81E-03**	0.24	2.07E-02	NS	**0.05**	**4.40E-02**	**2.58E-03**
rs4788668	72901874		0.14	2.63E-03	1.34E-01	0.01	9.04E-01	NS	0.07	1.67E-02	NS
rs4788671	72915085		-0.18	6.08E-04	3.28E-02	0.21	2.33E-02	NS	**0.04**	**1.21E-01**	**9.82E-03**
rs6499594	72917601		0.11	1.06E-01	NS	-0.11	1.39E-01	NS	-0.02	9.69E-01	NS
rs16971366	72919582		**-0.21**	**6.49E-05**	**3.76E-03**	-0.03	8.17E-01	NS	**-0.11**	**5.75E-04**	**3.16E-02**
rs9940310	72925982		0.20	1.51E-02	6.77E-01	-0.13	1.17E-01	NS	0.01	3.92E-01	NS
rs9925261	72927533		-0.13	8.54E-03	4.01E-01	-0.18	3.84E-02	NS	-0.16	2.96E-03	NS
rs16971384	72931085		**-0.17**	**2.08E-04**	**1.17E-02**	0.01	9.16E-01	NS	-0.07	2.13E-03	NS
rs4788482	72937079		**-0.25**	**5.08E-08**	**3.15E-06**	-0.13	3.96E-01	NS	**-0.18**	**3.77E-07**	**2.33E-05**
rs4788679	72956544		**-0.19**	**4.26E-05**	**2.51E-03**	-0.24	5.57E-02	NS	**-0.22**	**3.31E-05**	**1.99E-03**
rs9936884	72960230		-0.07	4.72E-01	NS	0.04	5.51E-01	NS	0.00	9.89E-01	NS
rs4788488	72960283		**-0.21**	**8.73E-06**	**5.32E-04**	0.01	8.43E-01	NS	**-0.08**	**1.29E-04**	**5.47E-03**
rs8055870	72972090		0.13	1.95E-02	8.40E-01	0.18	8.18E-02	NS	0.16	1.19E-02	NS
rs6499600	72979374		**-0.20**	**3.94E-05**	**2.37E-03**	-0.16	1.78E-02	9.24E-01	**-0.18**	**1.06E-05**	**6.49E-04**
rs13336412	72981949		**0.20**	**3.10E-04**	**1.71E-02**	0.08	2.71E-01	NS	**0.14**	**8.72E-04**	**4.71E-02**
rs2228200	72984668		0.12	4.29E-01	NS	0.42	1.35E-01	NS	0.29	2.23E-01	NS
rs2106258	72990553		**-0.26**	**3.61E-12**	**2.31E-10**	**-0.34**	**1.36E-11**	**8.69E-10**	**-0.31**	**2.56E-21**	**1.64E-19**
rs2157786	72991286		**-0.24**	**2.70E-09**	**1.70E-07**	**-0.34**	**1.25E-09**	**7.90E-08**	**-0.29**	**1.39E-16**	**8.79E-15**
rs10852515	72991660	Transcribed	**-0.25**	**7.47E-15**	**4.85E-13**	**-0.34**	**1.73E-18**	**1.12E-16**	**-0.30**	**9.61E-31**	**6.25E-29**
rs7193297	72993831		0.21	1.20E-03	6.35E-02	-0.18	1.47E-02	7.93E-01	**-0.01**	**2.86E-01**	**1.18E-02**
rs7404992	72994419		-0.13	1.84E-01	NS	-0.16	4.24E-02	NS	-0.14	4.57E-02	NS
rs756717	72996162		0.06	2.60E-01	NS	0.08	3.47E-01	NS	0.08	3.07E-01	NS
rs4788683	72997747		-0.05	3.47E-01	NS	-0.06	3.98E-01	NS	-0.06	4.12E-01	NS
rs9921395	73001957		-0.09	9.19E-02	NS	0.19	1.24E-02	6.84E-01	0.07	4.06E-01	NS
rs12445932	73004432		-0.16	1.19E-02	5.50E-01	0.13	2.92E-01	NS	0.00	1.72E-01	NS
rs7199343	73009024	Kawasaki GWAS	-0.02	6.91E-01	NS	0.34	1.63E-03	9.78E-02	0.18	1.66E-02	NS
rs11075954	73012164		-0.09	6.86E-02	NS	-0.20	1.27E-03	7.74E-02	**-0.15**	**9.01E-04**	**4.77E-02**
rs2040508	73012685		-0.02	8.31E-01	NS	-0.10	1.89E-01	NS	-0.06	4.48E-01	NS
rs16971456	73013036		0.18	3.22E-02	NS	0.13	1.47E-01	NS	0.15	3.00E-02	NS
rs9930445	73013482		0.08	1.90E-01	NS	0.17	7.62E-03	4.27E-01	0.13	1.09E-02	NS
rs4788684	73013633		0.02	7.12E-01	NS	-0.20	8.60E-04	5.33E-02	-0.11	9.32E-03	NS
rs16971464	73016143		0.28	1.78E-02	7.85E-01	-0.21	5.98E-01	NS	0.00	1.35E-01	NS
rs16971465	73017061		0.16	8.41E-02	NS	0.00	9.91E-01	NS	0.07	2.90E-01	NS
rs4788489	73017118		-0.05	3.29E-01	NS	-0.19	4.30E-03	2.45E-01	-0.13	1.07E-02	NS

rs16971474	73019004		0.00	9.49E-01	NS	0.17	3.25E-02	NS	0.10	1.38E-01	NS
rs11640106	73020116		0.03	5.69E-01	NS	0.26	2.84E-03	1.65E-01	0.16	1.20E-02	NS
rs1858800	73024276		-0.10	7.52E-02	NS	-0.17	7.02E-02	NS	-0.14	3.30E-02	NS
rs756720	73028921		0.10	1.39E-01	NS	0.20	3.97E-02	NS	0.16	3.43E-02	NS
rs7193343	73029160	AF GWAS	-0.05	4.35E-01	NS	-0.04	6.29E-01	NS	-0.05	6.28E-01	NS
rs11075958	73033869		0.13	1.25E-01	NS	0.48	2.63E-03	1.55E-01	0.33	2.97E-03	NS
rs8056528	73036633		0.06	3.91E-01	NS	0.05	5.19E-01	NS	0.05	5.26E-01	NS
rs719353	73042551		0.00	9.49E-01	NS	0.01	9.23E-01	NS	0.00	1.00E+00	NS
rs4788689	73049830		-0.03	7.29E-01	NS	0.14	1.22E-01	NS	0.07	4.67E-01	NS
rs2106261	73051620	AF GWAS	0.01	8.85E-01	NS	0.15	1.06E-01	NS	0.09	3.17E-01	NS
rs11863932	73053579		-0.13	8.88E-02	NS	0.11	4.62E-01	NS	0.01	5.09E-01	NS
rs1548373	73059861		0.09	1.45E-01	NS	0.00	9.98E-01	NS	0.04	4.26E-01	NS
rs4788692	73065656		0.05	6.25E-01	NS	-0.10	1.88E-01	NS	-0.04	6.62E-01	NS
rs12373097	73068515		-0.01	8.46E-01	NS	-0.09	2.39E-01	NS	-0.06	5.25E-01	NS
rs8057081	73068977		0.00	9.86E-01	NS	0.05	5.25E-01	NS	0.03	8.58E-01	NS
rs4788696	73070310		-0.12	3.41E-02	NS	-0.25	1.68E-01	NS	-0.19	3.52E-02	NS
rs8060701	73073289		0.09	3.91E-01	NS	-0.04	6.36E-01	NS	0.02	9.14E-01	NS
rs9940321	73073808		0.02	7.24E-01	NS	0.04	5.94E-01	NS	0.03	7.93E-01	NS
rs9940520	73074012		0.20	2.89E-02	NS	0.01	9.44E-01	NS	0.09	1.26E-01	NS
rs11641701	73079212		-0.12	2.17E-02	9.11E-01	0.02	8.08E-01	NS	-0.04	1.24E-01	NS
rs7204751	73079683		-0.09	2.49E-01	NS	-0.05	5.05E-01	NS	-0.07	3.86E-01	NS
rs4788697	73087494		-0.10	6.68E-02	NS	-0.01	8.77E-01	NS	-0.05	2.25E-01	NS
rs8052905	73097663		0.14	2.83E-01	NS	0.08	3.13E-01	NS	0.10	3.03E-01	NS
rs739414	73097956		-0.10	8.17E-02	NS	-0.15	5.47E-02	NS	-0.13	2.86E-02	NS
rs8051826	73102456		0.11	2.99E-01	NS	0.24	1.55E-02	8.24E-01	0.18	2.96E-02	NS

Table S5. Primer sequences for Sequenom genotyping assays. Genotyping was performed in multiplexes, W1-W5.

Multiplex	SNP	PCR primer 1	PCR primer 2	Amplicon Length	Extension primer sequence
W1	rs117951282	ACGTTGGATGGCTACAAGTCCTCAACACTC	ACGTTGGATGGGAAAAGTTAAGCACTCCC	100	GCACTCCCACTTTCA
W1	rs16971384	ACGTTGGATGGTGGTTGCTGTCTTTCCCTG	ACGTTGGATGTCAGTCCATTGAACTGGAGG	100	GCCCCATGATGGAAGA
W1	rs10852515	ACGTTGGATGAATATCAGTAGCTCCTGCGG	ACGTTGGATGTAATCACACACCTCGCACCG	100	TTGGTTTTTGGTTTGGT
W1	rs7199343	ACGTTGGATGCATGGATTCTGCTGGCACTC	ACGTTGGATGGCCCTGATAGGGTTTTAAGC	92	GCTTTGGAATTTGGAAGTC
W1	rs2157786	ACGTTGGATGCTCTAGGTGAGGATGACTAC	ACGTTGGATGCACCCTATTCACCATTCTCC	95	tcCCATTCTCCCTGGTTAAA
W1	rs740178	ACGTTGGATGTCCTCCTTGTCTTCCTCAAC	ACGTTGGATGACCTCCTGGCAATGGGAGAC	90	ATGGGAGACCCCACTCTGGC
W1	rs7193343	ACGTTGGATGAAATGTCGAGTCCTAATGGC	ACGTTGGATGGAGGGGAAAGTTTGAACAGC	89	GAAAGTTTGAACAGCTTGTTT
W1	rs16971474	ACGTTGGATGCCCACAGGCAAGATCTTGTC	ACGTTGGATGTAGGCAGACCCCATCATTTC	100	cGACCCCATCATTTCGAGATTA
W1	rs4788482	ACGTTGGATGTGTTCCTGCTGCGAGAGTTC	ACGTTGGATGAGGAACTGCTGGATTCTTGG	118	caTTAGCATGGGCCTTTCTCAA
W1	rs2228200	ACGTTGGATGCTGCACATGAACGTGGAGC	ACGTTGGATGAGAGCTTGCACTGGTATGAG	99	cTCACCGCCTTCCACTCGTCCTC
W1	rs2106261	ACGTTGGATGAGAGCAGTCTCTGGCACACT	ACGTTGGATGCACAGATAGAGCTCGTCCAG	100	ATAGAGCTCGTCCAGAGAATTGT
W1	rs9940520	ACGTTGGATGAAGCAACATAAGGCAGGCTC	ACGTTGGATGCAGTTCAGACTCTCTGTTAG	82	CAGACTCTCTGTTAGAGGCCTACT
W1	rs7193297	ACGTTGGATGAATGAGCGCCTCGCGGAGA	ACGTTGGATGCATTCGTTGCAGGTGACCTC	100	GTGACCTCCTTGCTGGCGGGCTCGG
W1	rs16971464	ACGTTGGATGCTCATTTGACCTCTAACAATC	ACGTTGGATGAGGAGTCACTCAGATGTTTG	120	tgAACCTGTAAAATCGAAAATATGA
W1	rs4788679	ACGTTGGATGGAAGAAAGCCAGGTACGAAG	ACGTTGGATGACCTACCTACAGCCTTATGG	84	CCTACCTACAGCCTTATGGAACGACC
W1	rs2266943	ACGTTGGATGTGGCTCTGTTTTGACTGGTG	ACGTTGGATGTTCCCAAAAGGCCATTCCAC	111	ccTCAATTTCAAGACACCTTGCTCCCT
W1	rs1476646	ACGTTGGATGCCCGGGAATACTTTCACATC	ACGTTGGATGTTCAGTAGCAACTCAGAGCG	119	CAGAGCGTCGTGCTCTCTTGTCTTTGT
W1	rs2106258	ACGTTGGATGAGCCTGAACAGAGCCAAATC	ACGTTGGATGTTAGATCCTGGTTAGCGGAG	101	tTTAGATCCTGGTTAGCGGAGCCTTCG
W1	rs8052905	ACGTTGGATGCCACTACATGCAGCTTCTTG	ACGTTGGATGACCAAAGTATTCCCCAGTCC	109	GGGAAAGCAAGATCCTGAGAAAGAATA
W1	rs8056528	ACGTTGGATGCTTGTGAATTAGCGAGAACC	ACGTTGGATGAAGCTAAGTAGCCTAAGGAC	97	agTTTTGTGAAGCATCTTTGCTGCTTTT
W2	rs11640106	ACGTTGGATGAAATCTGAAGGCCCTGAGTG	ACGTTGGATGAACAGACTGTCCTTCTCCAC	113	CTCACCCTGCCCCCA
W2	rs756720	ACGTTGGATGGCAGCATTGGACTAAATGGG	ACGTTGGATGTGTTTTCCTGATCACCAGGG	96	tATCCTCAGCCTTCCC
W2	rs12929452	ACGTTGGATGGCACAGGGTGTTATATTGGC	ACGTTGGATGTATAAGGAACACCATCCTGC	98	ACCATCCTGCCACTTA
W2	rs8051826	ACGTTGGATGAGGGTTGGAACCCATGTAAC	ACGTTGGATGAACTTCCCTAGTATGGCCAG	96	tGAGGCTGGAAAACCA
W2	rs12373097	ACGTTGGATGTACAACAGACTTACTGCTTC	ACGTTGGATGGAGAGGGTGAAACGAGCATT	106	CATTTCTAGAGGCTCCC
W2	rs739414	ACGTTGGATGTTTCCGAAAGAGCTGTCGTC	ACGTTGGATGGAGAACGAGCTTCCCAAATC	84	tcCCCAAATCCAACTCCA
W2	rs11641701	ACGTTGGATGTTCCAGCAGTCTGTAATCAC	ACGTTGGATGTGATGCTTGCTAGACACTCC	120	cccaGGCCTGGCTCATCTT
W2	rs9930445	ACGTTGGATGACCAGTGATGGCAATGTGTC	ACGTTGGATGTTTGCTTTGTTGAGGCACAG	100	cGTTGAGGCACAGATTAGGT
W2	rs4788683	ACGTTGGATGGGTGCAGTTATGACGAAGGG	ACGTTGGATGATTACACAGTGCGACTGTTC	104	tcccGTGCGACTGTTCATGTTT
W2	rs1858800	ACGTTGGATGTCTAAAGCCCATGTCTCAGG	ACGTTGGATGGATCAGTATGTGGTGATGGG	109	CACGATGTGTGAGACTGATAAC
W2	rs8057081	ACGTTGGATGTCGAGACAAGCCACATGTCAC	ACGTTGGATGTATTCCAGAGCTGCACCATC	98	tcGCCACCGACCCTTGTCACTAC

W2	rs9940321	ACGTTGGATGACTCAGATATCCAGGAGCAG	ACGTTGGATGTCACATTGCTTATACCGCTC	119	tcCCGCTCAAACTTATGACCAGTTC
W2	rs16971456	ACGTTGGATGAGTCCTAAGAGAGCGTGGAG	ACGTTGGATGGGATGATGGAGGTGGATTAG	115	ggcgGCTACATCTGTAAAAGAGTAAA
W2	rs9936884	ACGTTGGATGTCTGGCTTTTGTTACTTGGG	ACGTTGGATGTTTGCGTCTCTGTCTCTCTC	99	agagCACACTCTCACTCTATCCTGCCC
W2	rs6499594	ACGTTGGATGGACATTCCCTGATGAAGGAC	ACGTTGGATGTGACCTCTCTTTACCCTTGC	115	acAAAGCTTGCTGAAATTTACAATCCCT
W2	rs9940310	ACGTTGGATGTGAGTGCTTCCTATCAAGGG	ACGTTGGATGCACGTGATCAGTGAACACAG	98	tgacCACAGGACGTGTGCATGTCAGTAC
W3	rs11863932	ACGTTGGATGCCTGGAGATTGTGAGTCATC	ACGTTGGATGAGAAGGCTCTCTAGGAACGG	100	TGAGACGTTGCTTCC
W3	rs6499600	ACGTTGGATGTCCCACCAGCACCAACCATC	ACGTTGGATGAGATTTCGCCTGCATGCCCT	106	cGCCTGCGGTTCATTTCC
W3	rs16971366	ACGTTGGATGAACCCTTCAGAAATAACCCC	ACGTTGGATGAGATACCTGTAGGAGGTGAC	99	tcCTGGGGATGAGGTCCA
W3	rs4788489	ACGTTGGATGTTCCGCATTAATGCTCTTCC	ACGTTGGATGGCAACTTAATAACCACTGACC	101	tacCCACTGACCACCTTCT
W3	rs8060701	ACGTTGGATGTGGAATCACATACGCAGAGG	ACGTTGGATGAAAACAATGGCAGGTTCCCG	109	ttGCAGGTTCCCGGCCACT
W3	rs8058014	ACGTTGGATGGGGACAGTTGTAGAGCATTA	ACGTTGGATGTAACTAGCCACAGACAAGAG	118	AGCCACAGACAAGAGAAATCT
W3	rs9925261	ACGTTGGATGAATGGCTCTCAAAGTGGCAG	ACGTTGGATGCCACACTCTAGCTCATGTTG	99	cccgCTTACACTGCCATGAATT
W3	rs756717	ACGTTGGATGAAAGCCAATCTCCTCTCCAG	ACGTTGGATGTAAGAGGAGAACGGAGCAAG	115	cGCCCACCAATTAATTGGTAAA
W3	rs2040508	ACGTTGGATGTGGCTGGAGGACATCTTTAC	ACGTTGGATGTTGGGATCCCCTTCCAAATG	99	aAAATGTCAACAAACAAGAAAAG
W3	rs1548373	ACGTTGGATGGTGAGAGGTTGAGCTTTGTG	ACGTTGGATGCTGAGACTCAAACTTCACCC	96	gggaACAGGATTGAGACAAGTTAT
W3	rs719353	ACGTTGGATGGGGTGACTTTGCTTCTGAAA	ACGTTGGATGGGATGTTAAGTCTCTGTGCC	98	ggacGAAAATTTTATCTTGGGCATAT
W3	rs4788689	ACGTTGGATGCATTCTTCAAGGTGCCATGC	ACGTTGGATGTCCAGGACTGGGTTTTACAC	95	ttcaAGGACTGGGTTTTACACACCATA
W3	rs4788671	ACGTTGGATGCAGAACCTTCTGAACTGCTC	ACGTTGGATGTTCAAGTGCTTAATAGTGCC	98	ggatTGCTTAATAGTGCCATGGGGCTC
W3	rs4788692	ACGTTGGATGGAATGCATGAACCAGAACGC	ACGTTGGATGACAGTGGCCCTTCTTTTCAG	120	gaacGAGGGCCCGTGGTGACCTGCAGAG
W4	rs4788696	ACGTTGGATGAGCCATTTGCATTCCTCCAG	ACGTTGGATGTCCTAAACTCCTTTTCCCCC	99	TTCCCCCCACAAAGC
W4	rs11075958	ACGTTGGATGAGCTCTAAGAGCAGGGCAAC	ACGTTGGATGTGCACACCTTCTCTTGCCTG	117	ggGCCTGCAGCCAAAGC
W4	rs699444	ACGTTGGATGGCAGATTGGTCATTTGAGTG	ACGTTGGATGATCTGCAGGCAAATCTGGTG	94	ggATCTGGTGACAGCGG
W4	rs12445932	ACGTTGGATGTTGGTGAAAGGAGGTTCTGC	ACGTTGGATGTGACTGGTGCTCCTGACTTG	102	ccatCACGGATCCCCTCCC
W4	rs4788684	ACGTTGGATGGCCCCATAAGAATACCTAGC	ACGTTGGATGTTCTTCTTTTGCCCGGGCCT	118	cctttCCCGGGCCTCAATTT
W4	rs16971465	ACGTTGGATGTTTCTGCCATCACGGTGGAG	ACGTTGGATGGGTGGTCAGTGGTTATTAAG	115	cGTCTCATTGTTTTCTATCACG
W4	rs4788488	ACGTTGGATGGTGTTTGGAAAGTGGAGTCG	ACGTTGGATGCCCTTCCCAAGTAACAAAAG	96	GCCAGATAAATCCATGGAAGAG
W4	rs4788697	ACGTTGGATGGGAGGTGTTACAAAGAAAGG	ACGTTGGATGAAACACTCCGACATCCACTC	101	actaGCCCTGGTATTTTCATGGCA
W4	rs7204751	ACGTTGGATGGAAATGGAAAACAGTAGCTC	ACGTTGGATGGGTCACTTCCCTCTCTCAAC	115	CCCCCACTTTTTTTTTTTTTTTTGAA
W4	rs11075954	ACGTTGGATGTTCTGCTCTGGGATGGAAAG	ACGTTGGATGGCGTGTGAGAGGGTAATAGG	111	AGGAAATAAAAATAAAAACCCACAATTA
W5	rs13336412	ACGTTGGATGTTCCTTAGTGGCAAGAGAAG	ACGTTGGATGGGATTGTCATTCCAGTGCTC	100	CCCTTTGGTCCCTCA
W5	rs9921395	ACGTTGGATGCAACAATAACTTCACTGTGG	ACGTTGGATGTGCTGGTGATGGGAACTTTG	93	TCAATGAAGGTGGTGT
W5	rs4788668	ACGTTGGATGACAGAGTCTGCCAAGGAACC	ACGTTGGATGATCCCATTCGTCAGTGGCTC	106	GTGGCTCAGTTAGTTCC
W5	rs7404992	ACGTTGGATGGCGGGAATTTCCATTCTTTG	ACGTTGGATGTAGAGGGCTGGAGAGATGAC	99	CGTTCTCCACTGTGGCCC
W5	rs8055870	ACGTTGGATGAGCAGCTGGAGAGACAGTTC	ACGTTGGATGGGAAGAGCATATTTCCTGTC	98	TCTTTTATGCTCTAGTGACA

Table S6. Primer sequences for Sequenom allelic expression assays.

Multiplex	SNP	PCR primer 1	PCR primer 2	Amplicon Length	Extension primer sequence
W1	rs10852515	ACGTTGGATGAATATCAGTAGCTCCTGCGG	ACGTTGGATGTAATCACACACCTCGCACCG	100	TTGGTTTTTGGTTTGGT
W1	rs740178	ACGTTGGATGTCCTCCTTGTCTTCCTCAAC	ACGTTGGATGACCTCCTGGCAATGGGAGAC	90	ATGGGAGACCCCACTCTGGC

Supplementary Figures and Tables

Figure S1. Expression values.

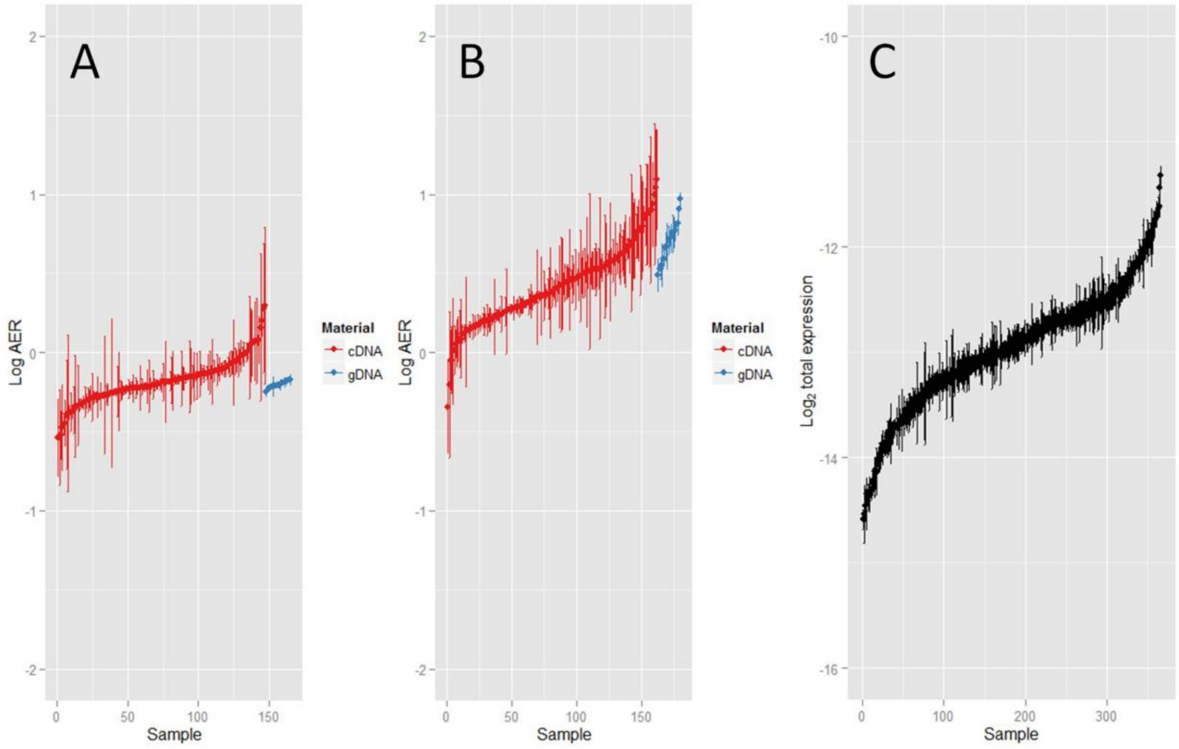

Panels A & B show log AER values plotted as the mean and standard error of four technical replicates on the y axis and rank on the x axis. AER values recorded in cDNA are shown in red with the AER of the genomic DNA samples used for assay normalisation shown in blue. For both transcribed SNPs rs740178 (A) and rs10852515 (B) the mean ratio for the normalisation samples differed from 0, demonstrating the need for a normalisation procedure. Panel C: normalised whole gene expression vales are shown. Values are plotted as the mean and standard error of four technical replicates on the y axis by rank on the x axis.

Figure S2. Linkage Disequilibrium in the NE and SA populations.

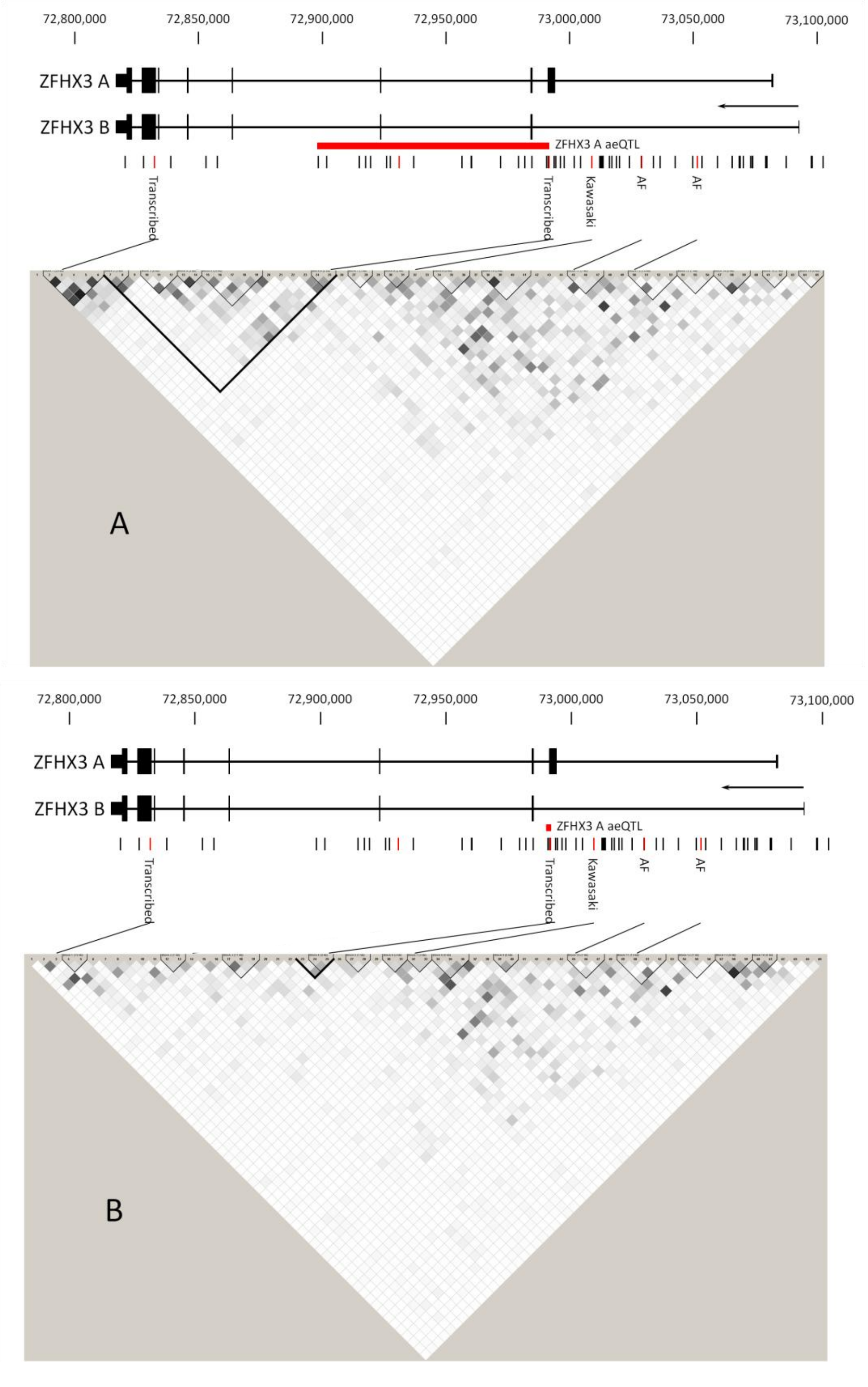

LD is shown for the NE population (Panel A) and the SA population (Panel B). Shading indicates r^2 Haplotype blocks are indicated by thin black triangles. The aeQTL for ZFHX3 A in each population is delimited by a thick black triangle and red bar. The cartoon represents the relative positions of the ZFHX3 isoforms, which are transcribed in the reverse direction, arrow.

Figure S3. Correlation between LD and strength of association.

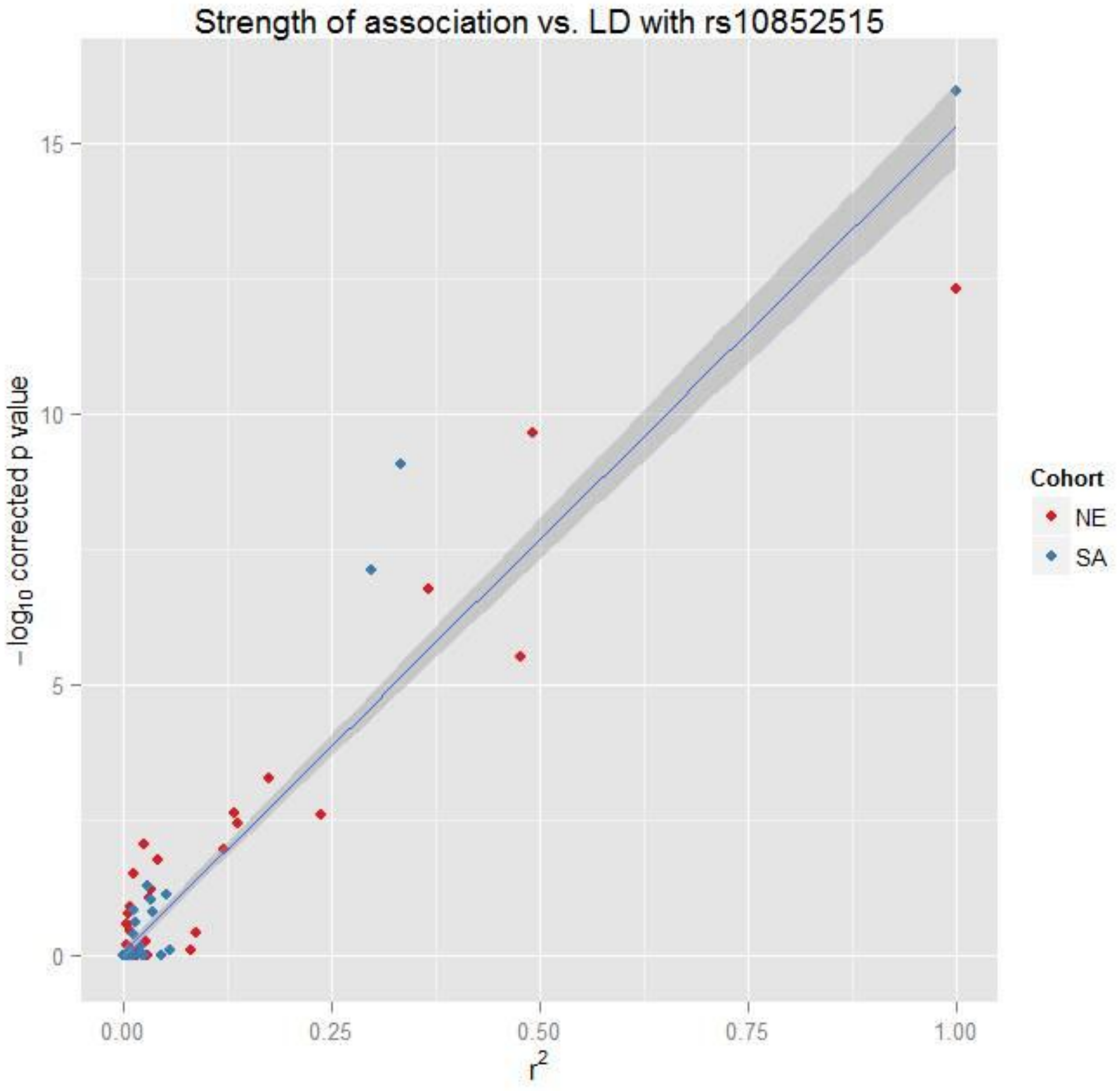

r^2 values are plotted against strength of association for SNPs in both cohorts. Colour indicates cohort.

The line represents a linear regression with 95% confidence intervals.
